# Fabrication of WO_3_ Quantum Dots with Different Emitting Colors and Their Utilization in Luminescent Woods

**DOI:** 10.3390/nano14110936

**Published:** 2024-05-27

**Authors:** Kwang Hyun Park, Nam Chul Kim, Sung Ho Song

**Affiliations:** Division of Advanced Materials Engineering, Center for Advanced Materials and Parts of Powders, Kongju National University, Cheonan-si 31080, Republic of Korea; recite14@kongju.ac.kr

**Keywords:** tungsten oxide quantum dots, quantum dots, photoluminescence, intercalation, exfoliation

## Abstract

With a rising interest in smart windows and optical displays, the utilization of metal oxides (MOs) has garnered significant attention owing to their high active sites, flexibility, and tunable electronic and optical properties. Despite these advantages, achieving precise tuning of optical properties in MOs-based quantum dots and their mass production remains a challenge. In this study, we present an easily scalable approach to generate WO_3_ quantum dots with diverse sizes through sequential insertion/exfoliation processes in solvents with suitable surface tension. Additionally, we utilized the prepared WO_3_ quantum dots in the fabrication of luminescent transparent wood via an impregnation process. These quantum dots manifested three distinct emitting colors: red, green, and blue. Through characterizations of the structural and optical properties of the WO_3_ quantum dots, we verified that quantum dots with sizes around 30 nm, 50 nm, and 70 nm showcase a monoclinic crystal structure with oxygen-related defect sites. Notably, as the size of the WO_3_ quantum dots decreased, the maximum emitting peak underwent a blue shift, with peaks observed at 407 nm (blue), 493 nm (green), and 676 nm (red) under excitation by a He-Cd laser (310 nm), respectively. Transparent woods infused with various WO_3_ quantum dots exhibited luminescence in blue/white emitting colors. These results suggest substantial potential in diverse applications, such as building materials and optoelectronics.

## 1. Introduction

Two-dimensional metal oxides (2D-MOs), characterized by diverse polymorphic crystal structures, have garnered significant attention due to their potential applications, including in smart windows, displays, and capacitors [1,2,3,4]. Tungsten trioxide (WO_3_) and perovskite quantum dots have garnered significant interest as compelling materials due to their outstanding luminescence, straightforward synthesis processes, and convenient tunability, particularly in the fields of photocatalysis, optoelectronics, and electrochromics [5]. Their appeal lies in the ability to tune electronic and optical properties through the manipulation of the crystal structure and chemical composition at edges or surfaces [6,7,8]. The optical properties of WO_3_ sheets have been documented within the range of 2.6–3.5 eV [9]. A wider band gap is generally advantageous, offering increased flexibility in optical sensing and imaging systems. This is attributed to the heightened sensitivity and resolution of devices associated with a broader band gap. A wider band gap enables more effective discrimination of specific wavelengths linked to particular materials or features. Despite these advantages, achieving a significant widening of band gaps remains a persistent challenge, even with the introduction of doping, alloying, strain engineering, and surface treatments.

Manipulating the size, crystal phase, and composition of tungsten oxide quantum dots imparts exclusive optical and electronic traits at the nanoscale. These characteristics offer advantages in tailoring materials to meet the specific requirements of diverse applications. Various techniques have been reported for tungsten oxide quantum dot fabrication. One method involves chemical precipitation, encompassing the formation of tungsten nanoparticles and their stabilization in a solution [10]. Hydrothermal methods, operating under high temperature and pressure conditions with the addition of chemicals (oleic acid or hydrazine hydrate), enable control over the size of the quantum dots [11,12]. Microemulsions, stable colloidal dispersions of oil and water stabilized by surfactants, serve as reaction media for synthesizing tungsten oxide quantum dots [13]. In this process, tungsten oxide precursors are introduced into the microemulsion system, leading to the formation of quantum dots. Electrochemical methods involve the reduction of tungsten ions on an electrode surface, allowing for controlled size and properties of tungsten oxide quantum dots through aliphatic amine chemicals in electrochemical conditions [6,14]. Additionally, UV light irradiation (365 nm) expedites the synthesis of tungsten oxide quantum dots, presenting enhanced thermal/photonic stabilities for blue PL emission [15]. Intercalation-based exfoliation methods are acknowledged as efficient strategies for producing metal oxide-based quantum dots, causing minimal damage to WO_3_ crystals. Notably, the selection of an appropriate solvent is pivotal in intercalation-based exfoliation and the subsequent processing of layered materials. A solvent with suitable surface tension interacts effectively with the material, facilitating the penetration of solvent molecules between layers. This interaction aids in overcoming interlayer forces, leading to successful exfoliation with tailored individual nanosheets or quantum dots of different sizes and thicknesses [4,16]. In this regard, understanding and controlling the surface tension of the solvent is essential for optimizing the exfoliation of layer-structured materials. This control ensures successful dispersion, stability, and size/thickness control of the exfoliated nanomaterials, impacting their properties and applications.

Here, we present a straightforward and efficient method for synthesizing WO_3_ quantum dots of varying sizes through the insertion of potassium ions and subsequent exfoliation in the solvents with different surface tension properties. The structural and optical properties of the WO_3_ quantum dots were comprehensively examined using high-resolution transmission electron microscopy (HR-TEM), atomic force microscopy (AFM), X-ray photoelectron spectroscopy (XPS), Raman spectroscopy, UV–vis absorption spectroscopy, and photoluminescence (PL). Additionally, quantum dots with different particle sizes were prepared and subjected to detailed analysis. The observed distinctions in WO_3_ quantum dots were attributed to the surface energy variations among the three solvents and the potassium-intercalated WO_3_ compound. Also, with the growing interest in environmentally friendly, energy-saving, and highly stable solid-state lighting solutions, particularly for applications in green and sustainable interior illumination and decoration, the luminescent woods with different emitting colors were fabricated by impregnation of the WO_3_ QDs in the delignified wood structure. These findings suggest significant potential across diverse applications such as smart windows and building materials.

## 2. Materials and Methods

### 2.1. Fabrication of WO_3_ Quantum Dots of Different Sizes and Luminescent Woods

WO_3_ powder, naphthalene, tetrahydrofuran (THF), N,N-dimethylformamide (DMF), N-methyl-2-pyrrolidone (NMP), and formamide were purchased from Sigma-Aldrich, St. Louis, MO, USA. The manufacturing process of WO_3_ intercalation compounds (WICs) was carried out in a vacuum system. After dissolving potassium metal in a solution in which naphthalene-THF was dissolved, WO_3_ powder was mixed with the solution. In more detail, a mixture was prepared by dispersing potassium metal (0.39 g) and naphthalene (1.28 g) in tetrahydrofuran (10 mL). Subsequently, WO_3_ powder (0.5 g) was added to the solution and maintained at 60 °C for 3 h. After the removal of naphthalene using cyclohexane, the intercalation compound powder, precipitated in cyclohexane, was introduced into the selected solvents (DI, THF, DMSO, ethanol, IPA, hexane, DMF, NMP, and formamide), followed by sonication for 1 h at temperatures ranging from 10 °C to 50 °C. This process aimed to tailor the fabrication of WO_3_ quantum dots (QDs) with varied sizes. The resulting samples, exfoliated in different solutions, were centrifuged at 1500 rpm for 30 min, yielding well-dispersed colloidal solutions named Red WO_3_ QDs (in formamide), green WO_3_ QDs (in NMP), and blue WO_3_ QDs (in DMF), respectively.

To fabricate transparent woods, Balsa wood with a thickness of 2 mm and a density of 0.26 g/cm^3^ was obtained from Midwest Products, USA. Sodium hydroxide (NaOH, 98.0%), sodium sulfite (Na_2_SO_3_, Daejung Reagents Chemicals, Daejung, Siheung, Gyeonggi-do, Republic of Korea), and hydrogen peroxide (H_2_O_2_, 30%, Daejung Reagents Chemicals, Republic of Korea) were also procured. Prior to the delignification and bleaching processes, each wood piece was dried at 100 °C. The dried wood pieces underwent delignification using a solution comprising sodium hydroxide (2.5 mol/L) and sodium sulfite (0.4 mol/L) dissolved in DI water. The woods were immersed in the lignin removal solution and boiled with stirring for 6 h. Following delignification, the delignified woods (DWs) were transferred to a bleaching solution (hydrogen peroxide mixed with DI water at a 1:1 weight ratio) to further eliminate lignin and other chemicals. The samples were bleached with stirring for 1 h. After the bleaching process, the woods were washed thrice with ethanol to remove any residual chemicals and then dried at 80 °C. Finally, luminescent woods were created by impregnating the delignified woods in WO_3_ quantum dot (QD) solutions. The delignified woods were immersed for 24 h at 30 °C and then dried for 24 h in an oven at 30 °C.

### 2.2. Characterizations

The dimensions of all samples were evaluated using atomic force microscopy (AFM; SPA400; SII, Chiba, Japan) in noncontact mode and high-resolution transmission electron microscopy (HR-TEM, Tecnai G2 F30, Hillsboro, OR, USA). Raman spectra, spanning from 200 to 900 cm^−1^, were obtained with a Raman spectrometer (LabRAM HR, Horiba, Palaiseau, France) employing 325 nm laser excitation. The chemical compositions were determined through photoelectron spectroscopy (XPS; Sigma Probe; AlK, Thermo Fisher Scientific, Kyoto, Japan). Photoluminescence (PL) measurements were conducted at room temperature using a 310 nm He-Cd continuous-wave (CW) laser, a mode-locked femtosecond-pulsed Ti:sapphire laser (Coherent, Chameleon Ultra II, Santa Clara, CA, USA), and monochromatic light from a 300 W Xenon lamp, respectively. We measured the QYs by using an absolute PL quantum yields measurement system (Hamamatsu Photonics, C9920-02G, Hamamatsu, Japan) that measured total photon flux by incorporating an integration sphere. UV spectrometers (Maya2000, Ocean Optics, Orlando, FL, USA) were used as a PL detector at room temperature.

## 3. Results

**Figure 1:**
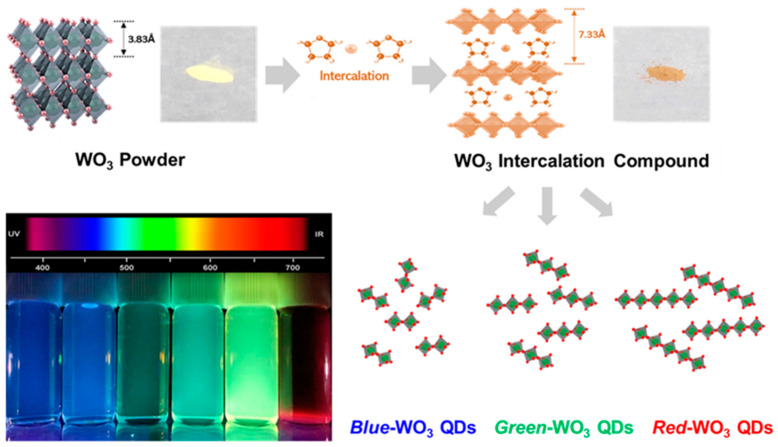
Schematic illustration for fabrication steps of WO_3_ quantum dots of different sizes.

In Figure 1, the sequential steps for synthesizing the WO_3_ quantum dots (QDs) of various sizes are depicted through an insertion and exfoliation process from bulk WO_3_ powder. Initially, WO_3_ intercalation compounds were synthesized by reacting WO_3_ powder with a mixture solution containing potassium metal (0.39 g) and naphthalene (1.28 g) in tetrahydrofuran (10 mL). Subsequently, the intercalation compounds underwent exfoliation in selected solvents with different surface tensions (DI (72 mN/m), THF (24.4 mN/m), DMSO (43.5 mN/m), ethanol (22 mN/m), IPA (23 mN/m), hexane (18 mN/m), DMF (37.1 mN/m), NMP (40.8 mN/m), and formamide (58.2 mN/m)) with sonication assistance, as shown in Figure S1. Following the intercalation process, the bulk WO_3_ powder, initially yellow in color, transformed into a light orange. Ultimately, three distinct WO_3_ quantum dots were fabricated, exhibiting blue-, green-, and red-emitting colors under the excitation of a 365 nm UV lamp.

**Figure 2:**
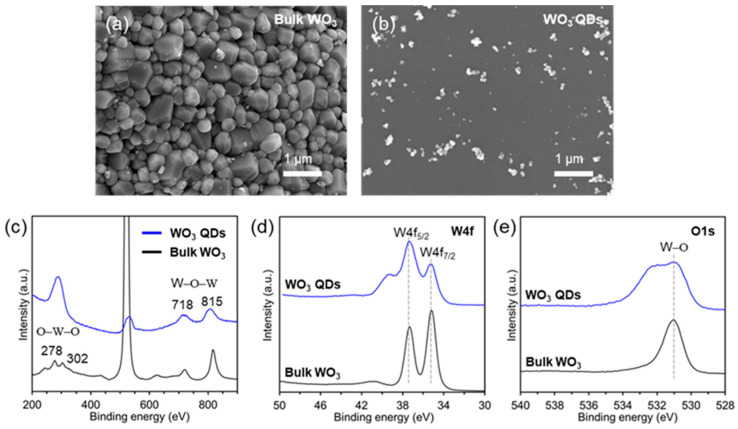
Characterizations of the WO_3_ QDs and bulk WO_3_ powder. (**a**) SEM images. (**b**) Bulk WO_3_ powder. (**c**) Raman spectroscopy and (**d**,**e**) chemical composition for tungsten and oxygen measured by XPS. (W4f spectra (**d**) and O1s spectra (**e**)).

The microstructural properties of both bulk WO_3_ powder and WO_3_ quantum dots (QDs) were assessed using scanning electron microscopy (SEM), and the outcomes are depicted in Figure 2a,b. The bulk WO_3_ exhibited an asymmetric surface particle morphology with a size of approximately ~1 μm and no discernible pores (Figure 2a). Following the exfoliation process to produce WO_3_ QDs, both the overall size and thickness were notably reduced to below 100 nm. Despite the similarity in SEM images for all the WO_3_ QDs, observed agglomeration during sampling is depicted in Figure 2b and Figure S2. These findings underscore that the morphological characteristics of the samples could be effectively altered through the exfoliation of WO_3_ intercalation compounds in the selected solvents. Raman spectroscopy and X-ray photoelectron spectroscopy (XPS) analyses were carried out to assess the crystal structure and chemical composition of the as-prepared WO_3_ QDs. The Raman spectra of the bulk WO_3_ powder exhibited four characteristic peaks (Figure 2c). The bands at 277 and 302 cm^−1^ were attributed to the bending modes of O−W−O, while the bands at 718 and 815 cm^−1^ in the high wavenumber region were assigned to the stretching modes of W−O−W [17]. Comparatively, the Raman spectra of the WO_3_ QDs revealed three characteristic peaks with lower intensity and broader peaks at similar locations compared to those of the bulk WO_3_ powder. Notably, the peaks observed at 277 cm^−1^ and 302 cm^−1^ disappeared, and a new peak emerged at 290 cm^−1^. These results indicate the successful fabrication of WO_3_ QDs, showcasing a reduction in both size and thickness compared to the bulk WO_3_ powder, aligning with the SEM analysis results.

To further investigate the composition of the nanosheets, X-ray Photoelectron Spectroscopy (XPS) characterizations were conducted. Figure 2d,e present the W4f and O1s spectra of both bulk WO_3_ and WO_3_ quantum dot (QD) samples. In Figure 2d, both bulk WO_3_ and WO_3_ QDs exhibit characteristic peaks related to the W4f core level, which split into two energy levels, W4f_7/2_ and W4f_5/2_. The peak positions for these energy levels were located at 35.2 and 37.3 eV, respectively. These positions align with the literature reports, indicating W^6+^ states in the WO_3_ QDs [16,17]. Furthermore, the O1s spectrum revealed a single peak that could be deconvoluted into two peaks at 532.1 eV and 530.8 eV. These peaks could be attributed to C-O and W-O, consistent with previous reports [6,16]. The XPS analysis results reaffirmed the presence of oxygen-rich groups on the surface of the WO_3_ QDs.

**Figure 3:**
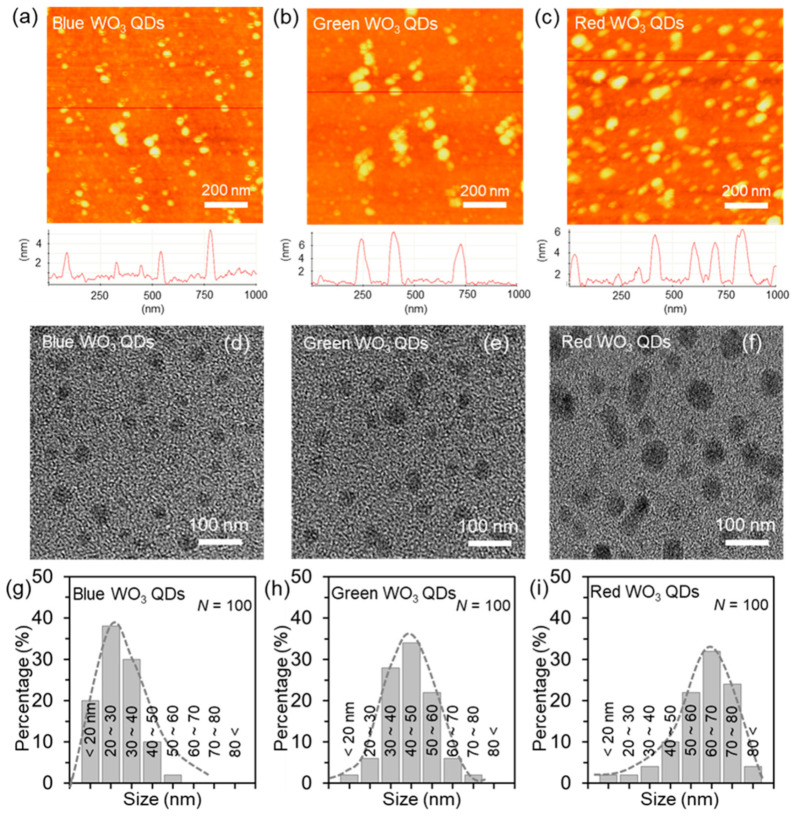
Characterizations of the WO_3_ QDs of different sizes. (**a**–**c**) AFM topology images (**top**) and thickness profiles (**bottom**) of the blue/green/red WO_3_ QDs. (**d**–**i**) TEM images of the blue/green/red WO_3_ QDs. Size distribution for (**g**) blue WO_3_ QDs, (**h**) green WO_3_ QDs, and (**i**) red WO_3_ QDs.

Atomic force microscopy (AFM) was employed for a detailed analysis of the thickness of the WO_3_ quantum dots (QDs) of various sizes. Figure 3a–c present representative AFM images, indicating that the thickness of all the WO_3_ QDs was below 6 nm, indicative of the few-layer structure of WO_3_. The lateral sizes for blue WO_3_ QDs, green WO_3_ QDs, and red WO_3_ QDs were approximately 30 nm, 50 nm, and 70 nm, respectively. Additionally, TEM images of individual WO_3_ QDs of different sizes were observed, as depicted in Figure 3d–f. Notably, all clear lattice spacing was measured at 0.37 nm (Figure S3), corresponding to the (002) plane of monoclinic WO_3_ [18]. To illustrate the size uniformity of the as-prepared WO_3_ QDs, a histogram depicting the size distribution was generated based on the results obtained from the TEM images (*N* = 100), as depicted in Figure 3g–i. The sizes of the blue WO_3_ QDs, green WO_3_ QDs, and red WO_3_ QDs were found to be in the ranges of 20–40 nm (accounting for ~88% of the distribution), 30–60 nm (86%), and 50–80 nm (~78%), respectively. Consequently, the average sizes of the three different WO_3_ QDs were approximately ~30 nm, ~50 nm, and ~70 nm, respectively. Additionally, Figure S3 presents the selected area electron diffraction (SAED) patterns of the representative WO_3_ quantum dots (QDs), revealing their single-crystal nature. We confirmed that the crystal lattice of the WO_3_ QDs, synthesized through an intercalation-based method, revealed a distinctive diffraction pattern consistent with a monoclinic structure and high crystallinity.

**Figure 4:**
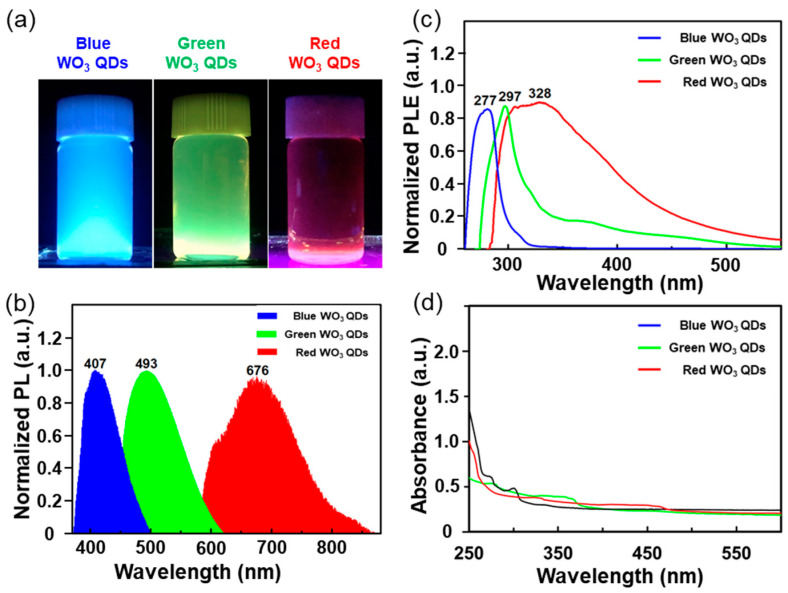
Optical characterizations of the blue WO_3_ QDs, green WO_3_ QDs, and red WO_3_ QDs. (**a**) Digital images of the WO_3_ QDs under excitation with a 365 nm UV lamp. (**b**) Normalized PL spectra of the WO_3_ QDs under excitation at 310 nm. (**c**) Normalized PLE spectra of the WO_3_3 QDs. (**d**) UV–vis spectra of the WO_3_ QDs.

Figure 4 illustrates the optical properties of the synthesized WO_3_ quantum dots (QDs) of varying sizes, as evaluated through photoluminescence (PL) and photoluminescence excitation (PLE) measurements. The emitted colors of the WO_3_ QDs, observed as blue, green, and red under UV lamp excitation at 365 nm, are depicted in the digital images of Figure 4a. The PL spectra, measured at the excitation of 310 nm, revealed maximum peak positions of 407 nm for blue WO_3_ QDs, 493 nm for green WO_3_ QDs, and 676 nm for red WO_3_ QDs, as shown in Figure 4b. Notably, the peak positions exhibited a consistent shift toward higher wavelengths with increasing size of the WO_3_ QDs, accompanied by peak broadening. The PL spectrum of the red WO_3_ QDs exhibited two types of shoulder peaks at approximately 630 nm and 800 nm. These results indicate that the emitting color, location, and range of the PL peaks were strongly influenced by the size and thickness of the WO_3_ QDs. Remarkably, the WO_3_ nanoparticles larger than the exciton Bohr radius exhibited photoluminescence with three distinct emitting colors, despite their sizes exceeding the exciton Bohr radius. These findings can be attributed to size-dependent properties arising from the formation of subdomains and functional groups on their surface or edges [19,20,21]. Additionally, PLE spectra were assessed at the maximum PL peaks, as depicted in Figure 4c. The maximum PLE peaks for blue, green, and red WO_3_ QDs were observed at 277 nm, 297 nm, and 328 nm, respectively. Furthermore, the maximum PLE peaks experienced a red shift with the increasing size of the WO_3_ QDs. All the WO_3_ dispersed in the solution exhibited light yellow colors, consistent with previous work [16]. Our as-prepared WO_3_ QDs displayed maximum peaks at various positions, accompanied by peak broadening, as shown in Figure 4d. Specifically, while bulk WO_3_ has been reported to exhibit a peak at 480 nm [22], the observed peaks for our WO_3_ QDs were situated differently. The blue-emitting WO_3_ QDs displayed peaks at around ~275 nm and ~300 nm, the green-emitting WO_3_ QDs showed a peak at around ~280 nm with broadening extending from 320 nm to 380 nm, and the red-emitting WO_3_ QDs exhibited broadened peaks spanning from 300 nm to 480 nm, respectively. These shifts in the absorption peaks could be attributed to the quantum size effect and surface states, which involved electron excitation from occupied orbitals to unoccupied anti-bonding components [23,24].

Furthermore, the quantum efficiency of the luminescence for the WO_3_ QDs with different emitting colors was analyzed using an absolute photoluminescence QY system, and the results are shown in Figure S4. This system quantified the total photon flux by integrating an integration sphere. The QYs for blue-emitting and green-emitting WO_3_ QDs were approximately 3.93% and 1.96%, respectively, whereas red-emitting WO_3_ QDs could not be measured. These results suggest that the red-emitting WO_3_ QDs possessed a wide size distribution, had low yield, and exhibited the formation of sub-domains with structural damages attributed to the stripping process, consequently reducing their quantum efficiency.

**Figure 5:**
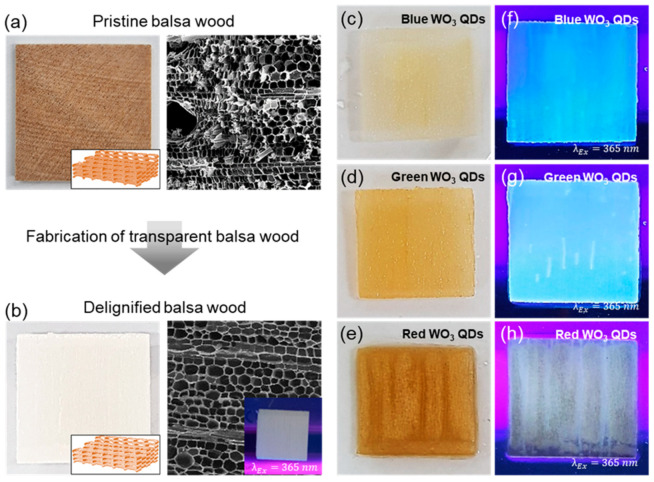
Digital and SEM images of pristine balsa, delignified balsa, and WO_3_ quantum dots (QDs) impregnated in the delignified balsa structure. (**a**) Digital (left) and SEM (right) images of the pristine balsa. (**b**) Digital (left) and SEM (right) images of the delignified balsa. Inset of SEM images: delignified balsa under excitation with a 365 nm UV lamp (**c**) (**d**,**e**) digital images of WO_3_ QDs impregnated in the delignified balsa for blue, green, and red QDs, respectively. (**f**–**h**) Digital images of WO_3_ QDs impregnated in the delignified balsa under excitation with a 365 nm UV lamp for blue, green, and red QDs, respectively.

As illustrated in Figure 5a,b, the spring-like lamellar structure of pristine balsa wood remained intact after the delignification process. The overall structure of the delignified balsa exhibited a well-preserved macrostructure with directional alignment of cellulose nanofibers. In Figure 1, top-view digital (left) and SEM (right) images were provided for both pristine balsa and delignified balsa. Following the delignification and bleaching processes of the pristine balsa wood, substantial changes were not observed in the macroscopic structure, which maintained a rectangular-/circular-like shape with thinner channel walls. However, a noticeable alteration occurred in the reduction of the thickness of the cell walls and a change in color, accompanied by a shift from light brown to white. These transformations were attributed to the extraction of lignin and hemicellulose. The delignified balsa wood was impregnated with different types of WO_3_ QDs to investigate the characteristics of luminescent transparent woods. When exposed to a 365 nm UV lamp, the delignified balsa did not exhibit any emitting color, as depicted in the inset of the SEM image in Figure 5b. The luminescent woods with different emitting colors were also successfully produced by embedding on the surface of the three-dimensional cellulose-based structure, while there was no change in the colors, as shown in Figure 5c–e. Under excitation with a 365 nm UV lamp, the luminescent woods infused with green WO_3_ QDs exhibited a white emitting color (Figure 5f), whereas the one infused with blue WO_3_ QDs displayed a vibrant blue color (Figure 5g). The luminescent wood fabricated with red WO_3_ QDs did not exhibit a distinct emitting color (Figure 5h). Unfortunately, the rationale behind the white-emitting characteristics resulting from the integration of green-emitting WO_3_ QDs remained unclear, likely due to changes in optical properties arising from physical and chemical interactions between cellulose-based wood and WO_3_ QDs, along with potential QD agglomeration. This result suggests that transparent luminescent woods incorporating cellulose and WO_3_ QDs can create a synergy between a light diffuser and a conversion layer. A noticeably enhanced light propagation parallel to the fiber direction was also observed in our work under in-plane excitation.

## 4. Conclusions

This study successfully achieved the creation of scalable WO_3_ quantum dots of different sizes through the exfoliation of the WO_3_ intercalation compound. The synthesis of tungsten oxide quantum dots demonstrated precise control over their size and surface properties using three solvents (DMF, NMP, and formamide). This control was facilitated by leveraging changes in wettability based on the difference in surface tension between WO_3_ and the solvent. The peeling process yielded WO_3_ units with quantum dot-sized dimensions, emitting R.G.B at 365 nm and presenting a notable dispersibility and exceptional fluorescence stability. Additionally, delignified woods with various fluorescence characteristics were created by immersing them in WO_3_ QDs with different emitting colors. The luminescent woods infused with green WO_3_ QDs exhibited a white emitting color, whereas the one infused with blue WO_3_ QDs displayed a vibrant blue color. Consequently, the intercalation-based exfoliation of quantum dots enabled size control through simple peeling and solvent manipulation, showcasing the potential for selective applications in various fields such as sensors and displays, tailored to user preferences for particle size, and extending into the electrochromic domain.

## Data Availability

Data are contained within the article and Supplementary Materials.

## Supplementary Materials

The following supporting information can be downloaded at https://www.mdpi.com/article/10.3390/nano14110936/s1: Figure S1. Digital images of the WO_3_ QDs exfoliated in various solvents with distinct surface tensions at temperatures of 10 °C and 50 °C, Figure S2. SEM images of blue/green/red WO_3_ QDs, Figure S3: HR-TEM images and the selected area electron diffraction (SAED) patterns of the representative WO_3_ quantum dots (QDs), Figure S4: Quantum yields of blue WO_3_ QDs and green WO_3_ QDs measured by using the absolute photoluminescence QY system.
