# Supplementary material for: Fabrication of WO3 Quantum Dots with Different Emitting Colors and Their Utilization in Luminescent Woods
Source: Nanomaterials (Basel). 2024 May 27;14(11):936. doi: 10.3390/nano14110936 (PMC11173498; doi:10.3390/nano14110936)
Supplement: Supplementary file 1 [file nanomaterials-14-00936-s001.zip › nanomaterials-2996174-supplementary.pdf]

Supplementary Materials

Fabrication of WO_3 quantum dots with different emitting colors and their utilization in luminescent woods

Kwang Hyun Park[a], Nam Chul Kim[a,*], and Sung Ho Song[a,*]

[a]Division of Advanced Materials Engineering, Center for Advanced Materials and Parts of Powders, Kongju National University, Cheonan-si 31080, Republic of Korea

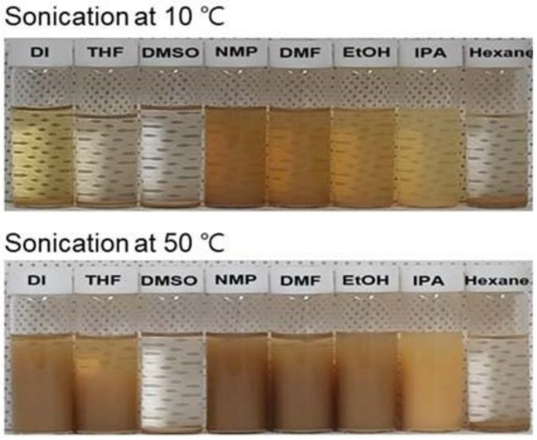

Solvents	Surface tension (mN/m)
DI	72
THF	24.4
DMSO	43.5
NMP	40.8
DMF	37.1
EtOH	22
IPA	23
Hexane	18

Figure S1. Digital images of the WO_3 QDs exfoliated in various solvents with distinct surface tensions at temperatures of 10°C and 50°C.

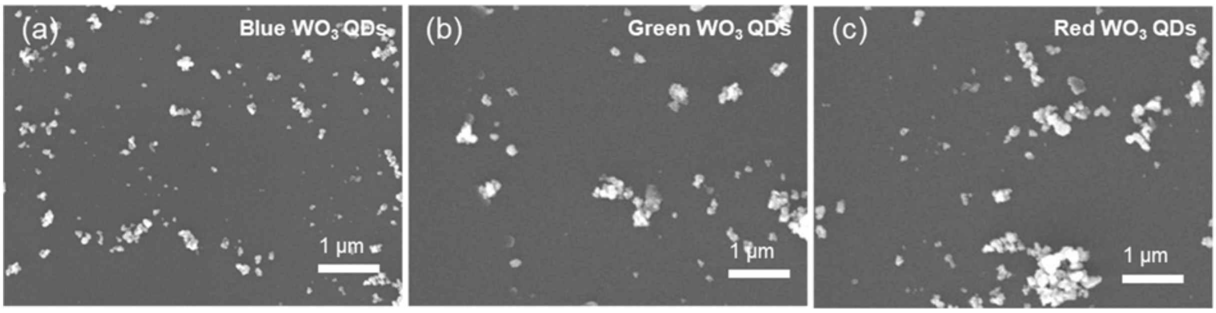

Figure S2. SEM images of Blue/Green/Red WO_3 QDs.

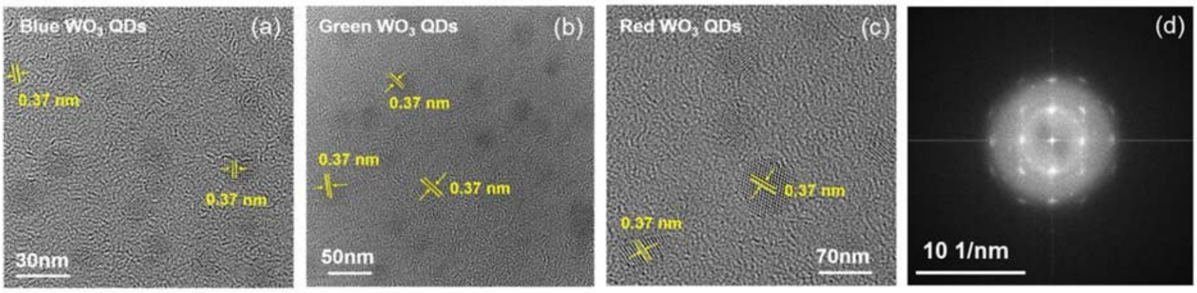

Figure S3. HR-TEM images and the selected area electron diffraction (SAED) patterns of the representative WO3 quantum dots (QDs)

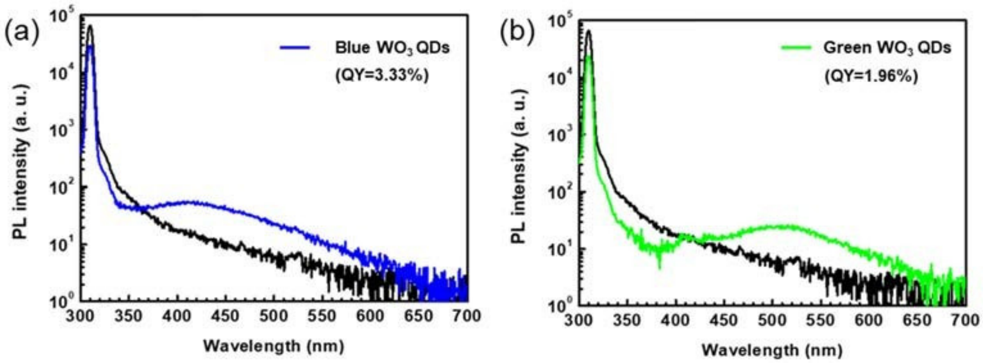

Figure S4. Quantum yields of blue WO_3 QD and Green WO_3 QD measured by using absolute photoluminescence QY system.
